# Simulation of a vacuum helmet to contain pathogen-bearing droplets in dental and otolaryngologic outpatient interventions

**DOI:** 10.1063/5.0036749

**Published:** 2021-01-12

**Authors:** Dongjie Jia, Jonathan Lee Baker, Anaïs Rameau, Mahdi Esmaily

**Affiliations:** 1 Sibley School of Mechanical and Aerospace Engineering, Cornell University, Ithaca, New York 14850, USA; 2 Brain and Mind Research Institute, Weill Cornell Medicine, New York, New York 10583, USA; 3 Sean Parker Institute for the Voice, Weill Cornell Medicine, Department of Otolaryngology - Head and Neck Surgery, New York, New York 10022, USA

## Abstract

Clinic encounters of dentists and otolaryngologists inherently expose these specialists to an enhanced risk of severe acute respiratory syndrome coronavirus 2 infection, thus threatening them, their patients, and their practices. In this study, we propose and simulate a helmet design that could be used by patients to minimize the transmission risk by retaining droplets created through coughing. The helmet has a port for accessing the mouth and nose and another port connected to a vacuum source to prevent droplets from exiting through the access port and contaminating the environment or clinical practitioners. We used computational fluid dynamics in conjunction with Lagrangian point-particle tracking to simulate droplet trajectories when a patient coughs while using this device. A range of droplet diameters and different operating conditions were simulated. The results show that 100% of the airborne droplets and 99.6% of all cough droplets are retained by the helmet.

## I. INTRODUCTION

Coronavirus disease 2019 (COVID-19), caused by severe acute respiratory syndrome coronavirus 2 (SARS-CoV-2), has rapidly evolved into a global pandemic.^1^ SARS-CoV-2 transmission occurs via direct and fomite contact, droplets, and aerosols.^2–8^ Due to the unique nature of the interventions performed by dentists and otolaryngologists, these specialists are at high risk of contracting the infection, becoming carriers of the disease and increasing the burden of health care systems.^9–11^ Routine outpatient interventions often elicit droplet-generating cough, sneeze, gagging, and dispersion of aerosols.^12–19^ The novel coronavirus is effectively threatening these specialists, their patients, and their practices.

The American Dental Association and the American Association of Otolaryngology—Head and Neck Surgery have recommended the avoidance of droplet and aerosol producing interventions on COVID-confirmed or suspected patients except in urgent cases.^20,21^ In communities with a high prevalence of COVID-19 infections, suspicion of infection should be assumed even in asymptomatic patients and proper isolation precautions have been recommended, including the performance of procedures in negative pressure or airborne infection isolation rooms, which are expensive, not required, nor available for all clinical encounters. Few, if any, of the outpatient clinics in dentistry and otolaryngology are currently fitted with negative pressure aeration. At Weill Cornell Medicine, infection control requires that one-hour elapses in non-negative pressure rooms used for aerosol-generating procedures before airborne isolation warnings are removed. Dentistry and otolaryngology rely on specialized diagnostic instruments, such as dental x-ray and nasal endoscopy, which are currently unavailable for telemedicine practice, limiting the ability to narrow differential diagnosis via online assessment. Although tele-practice has gained traction during the pandemic, it only functions as a screening tool and cannot replace the quality and depth of traditional in-person assessment and treatment until further technological advancement. With the expected progressive return to outpatient care, these specialists face substantial logistics challenges impacting the safety of their patients and the healthcare staff.

Current existing solutions are insufficient or cost-prohibitive for the safe management of office droplets and aerosols. For airborne generating procedures (AGPs), these include the use of N95 masks by all healthcare personnel and (1) airborne isolation measures for 60 min following APGs in non-negative pressure environments, which severely limits the number of patients who can be seen daily; (2) retrofitting standard rooms to a negative-pressure environment by exhausting indoor air directly outdoors or with the use of recirculating high-efficiency particulate air (HEPA) filter^22^ units to decrease turn-around time—a costly and often structurally infeasible modification;^23^ and (3) the use of surgical masks or modified surgical masks on patients undergoing AGPs. The surgical masks are worn until the procedure is started, which does not avert droplet and aerosol spread during the procedure. There have been attempts in otolaryngology to alter surgical masks via small perforation to allow the passage of a nasopharyngoscope and minimize the aerosol leakage with limited success in *ex vivo* models.^24^ However, these simple models do not account for the multiphase turbulent aerosols produced by sneeze and cough that propel droplets farther than in isolation, with clouds spanning up to 8 m.^25–27^

In this modeling study, we are proposing a novel open access vacuum helmet that would prevent the environmental release of pathogen-bearing droplets during outpatient AGPs in dental and otolaryngological clinics. Several studies have used numerical modeling to investigate droplet behaviors.^28,29^ We used computational fluid dynamics (CFD) in conjunction with a one-way coupled point-particle technique to simulate and evaluate coughing droplet movements when the helmet is in use. The simulated performance of the helmet design is presented and discussed.

## II. METHODS

In this section, the design of the vacuum helmet is first introduced and followed by the numerical approach for simulating the flow and droplets.

### A. Proposed design

**FIG. 1.**
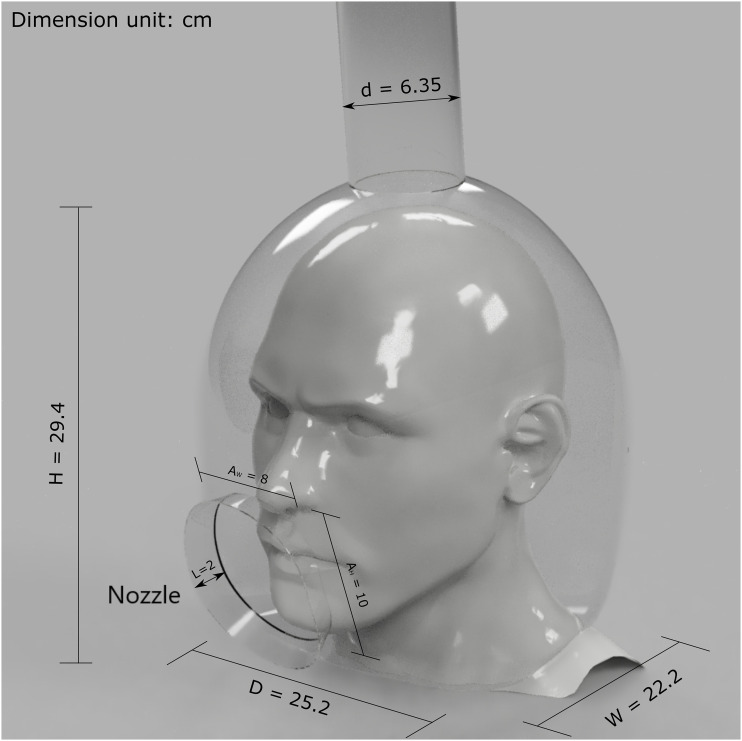
A visualization of the proposed helmet while in use with helmet dimensions shown in cm. D—helmet depth, W—helmet width, H—helmet height, L—nozzle size, d—vacuum port diameter, A_W_—access port width, and A_H_—access port height.

The proposed vacuum helmet design, which is shown in Fig. 1, has a shell that is designed to comfortably fit over the average patient’s head. This shell (1 mm thick) fully encloses the head with an access port and a vacuum port. The access port is a 10 × 8 cm^2^ opening on the helmet placed in front of the mouth and nose to provide access for medical procedures, while the vacuum port on top of the helmet, which is 2.5 in. (6.35 cm) in diameter, is connected to a vacuum source (e.g., negative air machine). The concept behind this design is to generate a reversal flow at the access port opening to carry away droplets to the vacuum port before they reach the environment. The vacuum source will contain a HEPA filter to ensure pathogen-bearing droplets are removed from the air prior to its recirculation to the environment. To improve the performance of this design, a nozzle is attached to the access port, which serves two functions. First, it extends the distance droplets must travel against the flow to minimize the probability of escape through the opening. Second, it allows for a smoother flow transition, thereby reducing patient discomfort generated by flow turbulence. The nozzle is 2 cm in length with a taper angle of 20°. To facilitate the use of different equipment in dental and otolaryngologic practices, we designed the spacing between the access port and the patient’s face to be as small as possible without any part of the face protruding outside of the opening. This choice produces a conservative estimate of the helmet performance, as the larger distance, in practice, translates to more effective capturing of expelled droplets by the helmet. The neck portion of the helmet is sealed with adjustable pads to ensure a tight seal. Since the flow rate requirement for the vacuum source of the helmet is small, it is much more affordable and accessible than a vacuum source required to create a negative-pressure room. For example, a typical medical-grade HEPA negative air machine, with a flow rate range of 150 CFM–600 CFM and a 99.99% HEPA filter efficiency for droplets ≥0.3 *μ*m, is mass-produced at a reasonable cost, thus allowing for the deployment of the proposed device at scale.^30^

### B. Simulation setup

The computational method used in this study was designed to ensure accuracy while keeping the computational cost affordable. Namely, the flow is fully resolved spatially using the finite element method (FEM), while droplets are modeled as point-particles using Lagrangian tracking. These formulations, as detailed below, are all implemented in our validated in-house Multiphysics finite element solver (MUPFES).^31–33^

**FIG. 2.**
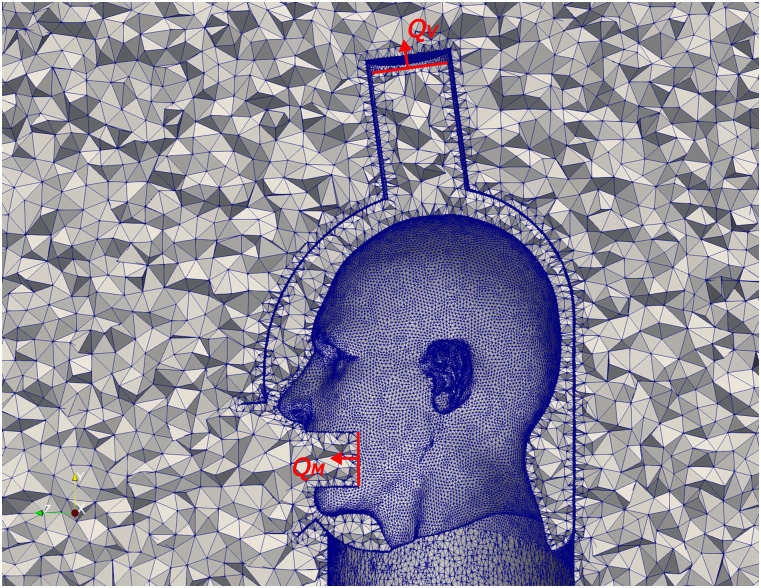
Cross-sectional view of the fluid field mesh. *Q*_*M*_—prescribed flow rate at the mouth intrusion and *Q*_*V*_—prescribed vacuum flow rate.

The flow is assumed to be incompressible since its Mach number is less than 0.07. The conservation of mass for fluid is satisfied by

$$\nabla \cdot u_{f}=0,$$ (1)

and the conservation of momentum for fluid is satisfied by

$$\frac{\partial }{\partial t}\left(\rho u_{f}\right)+\nabla \cdot \left(\rho u_{f}⊗u_{f}+pI-\mu \nabla u_{f}\right)=0,$$ (2)

where *ρ* = 1.225 kg/m^3^ is the air density, ***u***_f_(***x***, *t*) is the air velocity, *p*(***x***, *t*) is the air pressure, ***I*** is the identity tensor, and *μ* = 1.81 × 10^−5^ kg/m-s is the air dynamic viscosity. These parameters are taken for dry air at 15 °C, which do not change significantly if the temperature and humidity of the air vary in real life. To resolve the flow surrounding the helmet, we enclose it in a cube of 1 m^3^. To simulate the mouth opening during coughing, a cylindrical intrusion with a diameter of 3.5 cm and a depth of 4.0 cm is created at the mouth (Fig. 2). The cylindrical shape is an approximation, and the actual shape of mouth opening during coughing is more complex.^17^ The boundary conditions for Eq. (2) are no-slip ***u***_f_ = 0 for the helmet and head surfaces and constant *p* = 101.325 × 10^3^ Pa at the boundaries of the cubic enclosure. Provided the uncertainty surrounding the flow variation and epoch in which droplets are released during a cough, we model the worst-case-scenario in which the flow through the mouth opening is steady and equal to its peak value reported in the literature.^34^ Namely, we prescribe a constant flow of *Q*_*M*_ = 4.8 × 10^−3^ m^3^/s at the mouth intrusion. Two vacuum flow rates are simulated, which are *Q*_*V*_ = 70.8 × 10^−3^ m^3^/s (150 CFM) and 118.0 × 10^−3^ m^3^/s (250 CFM). The fluid field is meshed using Tetgen^35^ with tetrahedron elements and an edge length limit of 0.01 m, resulting in a mesh of 2.4 × 10^6^ elements. The cross-sectional view of the mesh as well as the boundary conditions imposed on the mouth and vacuum port interfaces is shown in Fig. 2. The flow is simulated for 2000 time points with a 10^−4^ s time step size to ensure steady-state conditions are achieved before droplets are introduced.

The droplet motion is modeled using a Lagrangian framework, in which

$${\dot{x}}_{p}=u_{p}$$ (3)

and

$$m_{p}{\dot{u}}_{p}=m_{p}g-f_{p},$$ (4)

where ***x***_p_(*t*) is the droplet position, ***u***_p_(*t*) is the droplet velocity, $\left(\dot{•}\right)$ denotes d(•)/d*t*, $m_{p}=\pi \rho_{p}d_{p}^{3}/6$ is the droplet mass, and ***f***_p_ is the force exerted on the droplet by the fluid. The droplet density *ρ*_p_ is 10^3^ kg/m^3^, and its viscosity *μ*_p_ is 8.9 × 10^−4^ Pa · s. The droplets are modeled as points with

$$f_{p}=3\pi \mu d_{p}C_{d}\left(u_{p}-u_{f}\left(x_{p},t\right)\right),$$ (5)

which accounts for the Stokes drag and neglects the Basset history terms in the Maxey–Riley equation.^36^ This assumption is justified by the high droplet-to-fluid density ratio (*ρ*_p_/*ρ* ≈ 810) and droplets being much smaller than the grid (i.e., smallest flow structures in the absence of droplets).^37^ Droplet–droplet interaction is inherently ignored by the point-particle model, which is justified by the alignment of droplet trajectories that results in a low effective volume fraction (*V*_p_/*V*_f_ ≈ 2.1 × 10^−4^). *C*_d_ in Eq. (5) accounts for the change in the Stokes drag at finite Reynolds number and is computed using an empirical relationship,^38^ that is,

$$C_{d}=1+0.15{Re}_{p}^{0.687},$$ (6)

in which Re_p_ = *ρ*‖***u***_p_ − ***u***_f_‖*d*_p_/*μ* is the droplet Reynolds number. The droplet Reynolds number in our simulations is of the order of 1. In Eq. (2), we neglected two-way coupling forces, given the mass loading ratio is ∼0.06 in these computations.

To compute drag from Eq. (5), the fluid velocity at the location of the droplet ***u***_f_(***x***_p_) is needed. Given that the grid is unstructured, one must identify the element that bounds individual droplets at each time step. To ensure the efficiency of this operation, we adopt an optimal particle localization scheme in our computations.^39^

Droplet evaporation is modeled based on empirical data^40^ obtained from droplet settling under gravity ***g*** as

$$\dot{d_{p}^{2}}=-10^{-10}\left(\frac{0.5\|a_{p}\|}{\left\|g\right\|}+1\right),$$ (7)

where ***a***_p_ is the droplet acceleration. Four initial cough droplet diameters are simulated: 200 *μ*m, 250 *μ*m, 500 *μ*m, and 1000 *μ*m. Given that the computations are one-way coupled and droplet and flow behaviors are independent of the number of simulated droplets, we simulated 100 droplets for each diameter class, which is sufficiently large to ensure statistical convergence. These 100 droplets are released with uniform spacing at the mouth intrusion surface after the flow field reaches a steady state. The initial velocity of each droplet is interpolated from the fluid velocity at its release location. The droplet simulation is continued for additional 2000 time points. Since the temporal resolution is 10^−4^ s, the droplets are tracked for 0.2 s that exceeds the peak velocity time (PVT), which is around 0.1 s, as reported in the literature.^34^

Before reporting the results, a mesh independence study was performed on the model. The simulation results did not change when the mesh element edge length limit is reduced by half, showing that our results are independent of the choice of spatial discretization.

## III. RESULTS

**FIG. 3.**
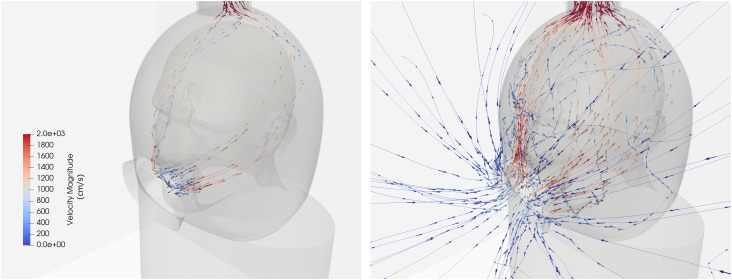
Velocity streamline passing through the mouse intrusion (left) and the helmet access port (right).

The flow streamlines for the 150 CFM vacuum flow rate are shown in Fig. 3 following the simulation convergence to steady-state, depicting the direction of the flow from the mouth and access port toward the vacuum port. The flow to the vacuum port is split between the mouth and the access port at 6.8% and 93.2%, respectively. This produces a mean velocity at the access port, that is, 9.4 m/s, and at the mouth opening, that is, 5.0 m/s. The access port accounting for a larger portion of the flow and operating at a higher velocity than the mouth opening is the precursors for the effective operation of this helmet, as confirmed by our droplet simulation results summarized in Table I. In Table I, the percentage of droplets in each diameter class as well as those that escaped from the helmet are reported. As elaborated in Sec. IV, if droplets of a certain diameter are fully captured, all droplets smaller than such size will be captured. Thus, we grouped all droplets ≤200 *μ*m in size as a part of the same class. The distribution of droplets reported in Table I is based on the experimental measurements performed on coughing subjects.^41^ The percentage of total droplets escaped is calculated by multiplying the percentage of droplets escaped for a certain diameter to the percentage of such diameter droplets among all droplets emitted. Overall, more than 99.6% of the droplets are captured by the proposed device for both 150 CFM and 250 CFM vacuum flow rates.

**TABLE I.** Results of droplet simulations with vacuum flow rates of 150 CFM and 250 CFM.

Vacuum flow rate (CFM)	150				250			
Droplet diameter (*μ*m)	≤200	250	500	1000	≤200	250	500	1000
Percentage escaped for each diameter (%)	0	0	27	46	0	0	16	43
Percentage distribution of droplets^41^ (%)	98.46	0.58	0.68	0.28	98.46	0.58	0.68	0.28
Percentage of total droplets escaped (%)	0.00	0.00	0.18	0.13	0.00	0.00	0.11	0.12

**FIG. 4.**
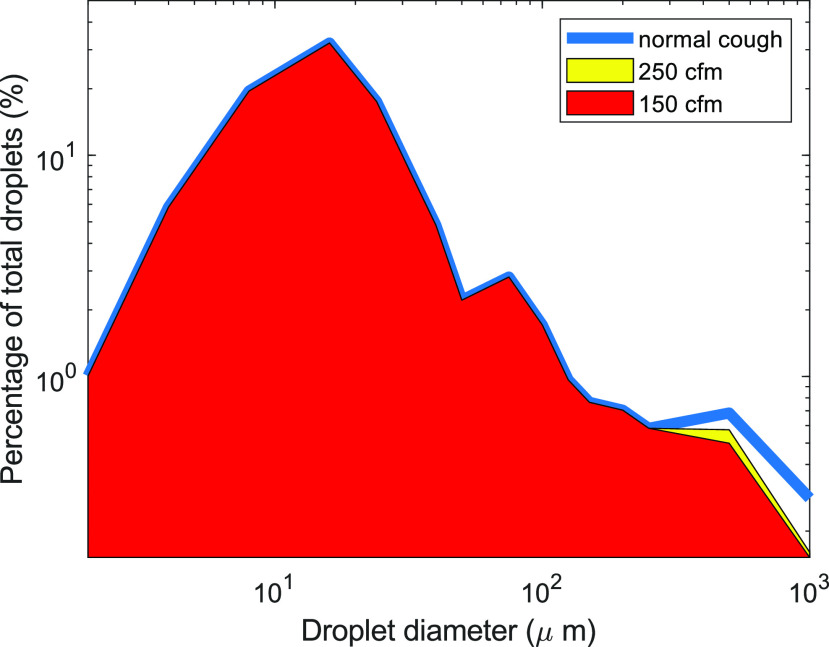
Helmet performance in capturing droplets during coughing when the vacuum source is operating at 150 CFM (red) and 250 (yellow) CFM. The blue line represents the distribution of released droplets from coughing. The shaded areas represent contained droplets. All droplets with a diameter ≤250 *μ*m are captured.

**FIG. 5.**
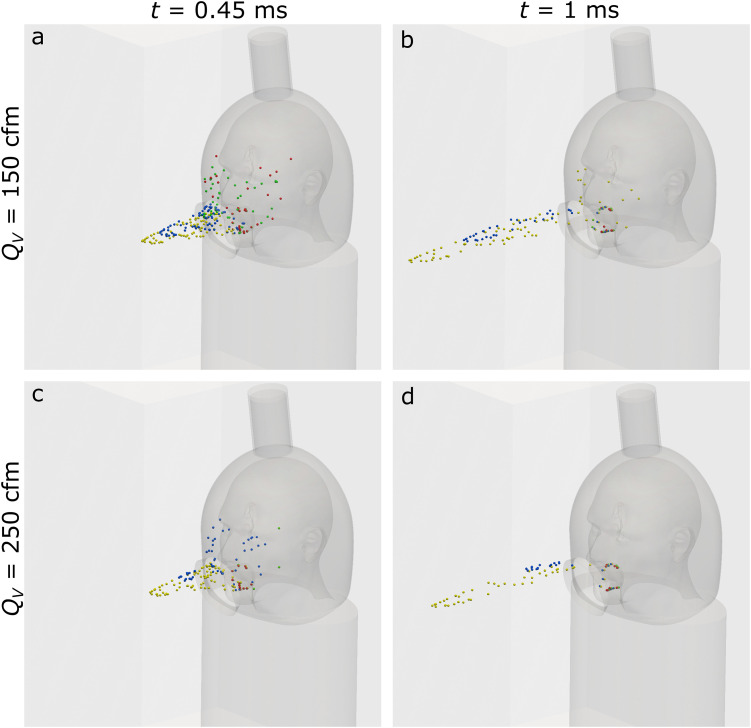
Droplet behaviors for 150 CFM [(a) and (b)] and 250 [(c) and (d)] CFM at 0.45 ms [(a) and (c)] and 1 ms [(b) and (d)]. Droplet diameters marked by color: 200 *μ*m (red), 250 *μ*m (green), 500 *μ*m (blue), and 1000 *μ*m (yellow). *Q*_*v*_—vacuum flow rate and *t*—time after droplets are released. Fewer droplets are visible at later time as they have exited through the vacuum port.

The effectiveness of the helmet in retaining droplets is further visualized in Fig. 4. The blue line is the droplet size distribution taken from the literature.^41^ The shaded areas represent the droplets that are retained by the helmet during vacuum conditions. The trajectories of the droplets in the simulation are shown in Fig. 5. Snapshots are taken after 0.45 ms and 1 ms after the droplets are released from the mouse intrusion surface. As the result shows, the droplets take around 0.1 s to either contain or escape through the opening port, while the duration of one cough is around 0.5 s. Therefore, repetitive coughing can be considered as independent events and will not change the conclusions of this study.

## IV. DISCUSSION

Whether a droplet follows a ballistic trajectory or acts as a neutral tracer can be quantified using a non-dimensional parameter called Stokes number, which is the ratio of the particle relaxation time (which itself depends on the particle diameter and density and fluid viscosity) to the flow time scale. For the cases modeled in this study, where all parameters are held fixed except for the particle diameter, the relative Stokes number between modeled droplet classes varies proportionally to their diameter squared. This relationship demonstrates that the time that it takes for a droplet of 1000 *μ*m to respond to the background flow is four times longer than that of a 500 *μ*m droplet. This quadratic change in response time translates to an equally longer travel distance for droplets before their trajectory being reverted by the reversal flow through the access port. This analysis explains the observed trend where the larger droplets were more likely to escape through the access port than the smaller ones.

The direct relationship between the droplet diameter and its ballistic behavior suggests that there is a cutoff limit below which all droplets are captured and above which it is progressively harder for the modeled device to retain droplets. The droplets smaller than this cutoff limit travel to a shorter distance against the flow than those at the cutoff limit. Thus, they will be sucked back into the helmet earlier with no chance of escaping through the access port. Although our analysis indicates the existence of this cutoff limit, its quantification relied on predicting the particle-laden flow dynamics surrounding the mouth opening using point-particle CFD simulations as previously conducted. We also plan to experimentally test this hypothesis in the near future.

Our simulations showed that the cutoff limit for the proposed design is ∼250 *μ*m. These results also confirmed that the higher the vacuum flow rate, the larger the cutoff limit will be, as there were fewer 500 *μ*m droplets escaping at 250 CFM than 150 CFM. Nevertheless, one can perform a pen-and-paper analysis to show that the rate at which this cutoff limit increases is proportional to the root square of the vacuum flow rate, requiring a flow rate four times larger to contain droplets that are only twice larger. Since a very large vacuum flow rate leads to moderate gain in effectiveness while producing significant patient discomfort, we consider 150 CFM to be the optimal operating regime of the proposed device.

Even though a large percentage of very large droplets escape through the helmet at 150 CFM, the proposed device can still significantly reduce the risk of transmission through expelled droplets for the following reasons. First, the large droplets escaping the helmet account for less than 1% of all droplets, leading to over 99.6% of all droplets being captured by the proposed device. Second, droplets that are smaller than 100 *μ*m in diameter pose the highest risk of transmission by forming “droplet-nuclei,” which remain airborne for hours or even days. All of these droplets are captured by our device. Third and most important, droplets that are over 100 *μ*m pose a lower risk as they fall to the ground from a stand-up position (2 m) within seconds after their release.^41^ Nevertheless, these very large droplets can create a risk if the clinical practitioner is positioned directly in front of the patient. As a mitigation strategy for these situations, the practitioner should be advised to wear a face shield as a primary barrier against the direct transmission of very large droplets.

In the future, we plan to optimize the nozzle shape at the face opening for optimal droplet containment. A longer nozzle will perform better in retaining the larger droplets since the exposure time for the opposing flow will be longer. However, a longer nozzle would reduce the procedural space used by the clinician, thus limiting the helmets general utility. A lower vacuum flow rate can be considered since more suction means more discomfort for the patient. Overall, finding a helmet design that successfully contains close-to-all droplets while providing comfort and usability for patients and clinicians is most desired. Our reported simulation predictions must be thoroughly validated experimentally^42^ before this device is deployed in practice. The performance of the helmet during dental procedures and sneezing also needs to be studied using both simulations and experiments. However, given that the peak air velocity through the mouth during sneezing is similar to that of coughing, we do not expect a significant difference in the performance of the proposed device during sneezing.^43,44^

The helmet shell would be contaminated by presumably infectious droplets; therefore, the helmets must be thoroughly disinfected or disposed of upon use. Polymethyl methacrylate (PMMA) plastic is an ideal choice for manufacturing the helmet shell since the material and shaping costs are low. The PMMA plastic is also transparent, therefore facilitating patient–doctor interaction as well as minimizing potential claustrophobia. The PMMA plastic is also among the strongest in its class so that the practitioners can rest their hands on the face of the helmet without compromising the structural integrity of the device.^45^

## V. CONCLUSION

Here, we proposed novel personal protective equipment (PPE) designed for containing pathogen-bearing droplets generated during violent respiratory events while providing access to the mouth and nose for clinical operations. We performed one-way coupled point-particle CFD simulations to examine the effectiveness of this device design. The model predictions show that the design can successfully contain all droplets less or equal to 250 *μ*m in diameter, which translates to over 99.6% of droplets generated from coughing. Future studies will build on this simulation study to improve the helmet design using formal shape optimization, validate the simulation results by fabricating a prototype and performing experiments, and investigate manufacturing processes for the mass production of the helmet.

## DATA AVAILABILITY

The data that support the findings of this study are available from the corresponding author upon reasonable request.
